# Pathways to diagnosis for Black men and White men found to have prostate cancer: the PROCESS cohort study

**DOI:** 10.1038/sj.bjc.6604670

**Published:** 2008-09-16

**Authors:** C Metcalfe, S Evans, F Ibrahim, B Patel, K Anson, F Chinegwundoh, C Corbishley, D Gillatt, R Kirby, G Muir, V Nargund, R Popert, R Persad, Y Ben-Shlomo

**Affiliations:** 1 Department of Social Medicine, University of Bristol, Canynge Hall, Whiteladies Road, Bristol BS8 2PR, UK; 2 Department of Urology, United Bristol Healthcare Trust, Bristol Royal Infirmary, Upper Maudlin Street, Bristol BS2 8HW, UK; 3 Department of Urology and Pathology, St. George's Hospital, Blackshaw Road, Tooting, London SW17 0QT, UK; 4 Department of Urology, Barts and the London NHS Trust, St Bartholomew's Hospital, West Smithfield, London EC1A 7BE, UK; 5 Department of Urology, Newham University Hospital NHS Trust, Glen Road, London E13 8SL, UK; 6 Department of Urology, Southmead Hospital, Southmead Road, Bristol BS10 5NB, UK; 7 King's College Hospital NHS Trust, Department of Urology, Denmark Hill, London SE5 9RS, UK; 8 Department of Urology, Guy's and St Thomas’ NHS Foundation Trust, Guy's Hospital, Great Maze Pond, London SE1 9RT, UK

**Keywords:** prostate cancer, ethnicity, access to health care

## Abstract

Black men in England have three times the age-adjusted incidence of diagnosed prostate cancer as compared with their White counterparts. This population-based retrospective cohort study is the first UK-based investigation of whether access to diagnostic services underlies the association between race and prostate cancer. Prostate cancer was ascertained using multiple sources including hospital records. Race and factors that may influence prostate cancer diagnosis were assessed by questionnaire and hospital records review. We found that Black men were diagnosed an average of 5.1 years younger as compared with White men (*P*<0.001). Men of both races were comparable in their knowledge of prostate cancer, in the delays reported before presentation, and in their experience of co-morbidity and symptoms. Black men were more likely to be referred for diagnostic investigation by a hospital department (*P*=0.013), although general practitioners referred the large majority of men. Prostate-specific antigen levels were comparable at diagnosis, although Black men had higher levels when compared with same-age White men (*P*<0.001). In conclusion, we found no evidence of Black men having poorer access to diagnostic services. Differences in the run-up to diagnosis are modest and seem insufficient to explain the higher rate of prostate cancer diagnosis in Black men.

Radical treatment can cure localised prostate cancer, but can lead to serious side effects, and many untreated men do not develop advanced disease (Albertsen *et al*, 2005). Risk factors and prognostic factors are needed to identify those men with an increased chance of developing cancer and of seeing that cancer progress, as these men have a more favourable balance of risks and benefits when undergoing screening tests and radical treatment. Black race is one of very few factors supported by convincing evidence (Gronberg, 2003). Surveillance, Epidemiology, and End Results (SEER) data from the United States indicate that Black men are 2.4 times more likely to die of prostate cancer than White men of the same age (US Cancer Statistics Working Group, 2005). In England, where prostate-specific antigen (PSA)-based screening is uncommon, the **PR**ostate **C**ancer in **E**thnic **S**ubgroup**S** (PROCESS) study has demonstrated Black men to have 3.1 times the incidence of prostate cancer as compared with their same-age White counterparts (Ben-Shlomo *et al*, 2008). Black men also have a poorer prognosis than similar age White men. A meta-analysis of US data found the disease-specific case-fatality rate to be 29% higher (Evans *et al*, 2008).

The higher rate of prostate cancer in Black men may arise from a genuinely higher incidence, or alternatively there may be an equal incidence of prostate cancer but a greater likelihood of diagnosis among Black men. US studies suggest that Black men have worse access to health care generally (Institute of Medicine, 2001), and may have poorer access to PSA testing in particular (Freedland and Isaacs, 2005), although a recent study has found Black men aged 40–49 years to be subject to a higher rate of testing (Ross *et al*, 2008). If Black men follow a different pathway to diagnosis compared with White men, the relative incidence of prostate cancer in US Black men may be obscured, making the role of disease biology difficult to investigate. As the UK National Health Service has an ethos of health-care equity (Whitehead, 1994), it is possible that differences in the diagnostic pathways followed by Black men and White men are avoided, in which case the higher incidence of prostate cancer in Black men in the UK PROCESS cohort could be more confidently attributed to a biological mechanism. Consequently our objective, in this study of the PROCESS cohort, is the first UK-based investigation of the pathways to prostate cancer diagnosis followed by Black men and White men in terms of demographic measures, knowledge concerning prostate cancer, time from symptoms to presentation and the reasons for delayed presentation, symptoms at presentation, referral pathways to diagnosis, and PSA level at diagnosis.

## Materials and methods

PROCESS, a population-based retrospective cohort study, has been described earlier (Ben-Shlomo *et al*, 2008). Males resident in four study areas (North Bristol, South West London, South East London, and North East London) were observed during 1995–1999 (Bristol) or 1997–2001 (London). Cases of prostate cancer were ascertained from the following sequence of sources: (a) pathology databases and (North West London only) a urology department database, (b) hospital discharge diagnosis files, (c) PSA records >10 ng ml^−1^, and (d) Cancer Registry (Bristol only). Where there was uncertainty, a panel of at least four urologists classified a case vignette as a ‘clinical’ (non-histological proven) case of prostate cancer, or excluded it due to a lack of evidence.

Men known to be alive were asked to complete a questionnaire including the 2001 census questions on ethnicity, with the next of kin being contacted if the man had died more than 6 months ago. Questionnaire information determined ethnicity for 37% of Black men and 45% of White men. If a man's ethnicity remained undetermined, we referred in turn to hospital records (62% of Black men, 50% of White men) and place of birth recorded on the death certificate (1% of Black men, 5% White men). Consultants recalled ethnicity for six men without other information.

The questionnaire also assessed demographic information, knowledge of prostate cancer, and delays in presenting with symptoms. If the questionnaire was not completed, occupation would be obtained from hospital records or the death certificate. Occupations were classified as manual or non-manual according to the Registrar General's scheme (see http://www.statistics.gov.uk/). Postcodes were linked to 1998 electoral wards (http://www.edina.ac.uk/), then to the Index of Multiple Deprivation (IMD) score for the year 2000 (http://www.neighbourhood.stati stics.gov.uk/). There are six domains (income; employment; health deprivation and disability; education, skills and training; housing; and geographical access to services) that determine the index score for an area, higher scores indicating greater deprivation.

Trained research nurses reviewed hospital records using a standard proforma (copies available from the authors), extracting information on delays in presentation, the presence of co-morbid illnesses contributing to the Charlson Index (Charlson *et al*, 1987), symptoms at presentation, diagnosis in the absence of symptoms, and referral pathways. To calculate the Charlson index for a man, each of his co-morbid conditions was assigned a weight (e.g., 1=myocardial infarct, 2=leukaemia, 3=moderate or severe liver disease, and 6=AIDS) and the weights were added together (Charlson *et al*, 1987). Although three centres completed reviews for more than 85% of cases, time constraints restricted attention to a sample of cases (70% of Black men and 42% of White men) at the North East London centre. In addition, an attempt was made to retrieve a measurement of each man's PSA level from around the time of diagnosis and prior to any treatment.

The South West Multi-Centre Research Ethics Committee approved the PROCESS study.

### Statistical analysis

Multivariable regression models estimated the associations between race and binary factors (logistic regression), unordered categorical factors (multinomial regression), and ordered categorical factors (ordered logistic regression) (Kirkwood and Sterne, 2003). Analyses were centre and age adjusted by including each of these covariates in regression models as four dummy variables, distinguishing the five study centres and five age categories. CI denotes confidence interval, and *P*-values are two-tailed throughout. Analyses were undertaken using Stata version 10 (StataCorp, TX, USA, 2007).

## Results

Reviews of hospital records were available for 87% (474 out of 547) of Black men and 75% (993 out of 1319) of White men; questionnaire data for 38% (210) of Black men and 46% (601) of White men. A greater proportion of Black men had completed the questionnaire themselves (193, 92%) as compared with the White men (463, 77%).

Black men presented at a younger age (mean: 67.9 years, s.d.: 7.3 years, *n*=547) compared with White men (mean: 73.3 years, s.d.: 8.8 years, *n*=1319), resulting in a centre-adjusted difference of 5.1 years (95% CI: 4.2–5.9 years, *P*<0.001). Black men lived in slightly poorer neighbourhoods (mean IMD score: 44.6, s.d.: 14.4, *n*=546) compared with White men (mean IMD score: 41.7, s.d.: 17.0, *n*=1311), an age- and centre-adjusted difference of 1.45 (95% CI: 0.34–2.56, *P*=0.010). More Black men (81%; 333 out of 413) than White men (67%; 739 out of 1110) were in manual occupations (*P*<0.001 age- and centre-adjusted).

The questionnaire assessed men's knowledge of prostate cancer, the responses in Table 1 being taken from those questionnaires completed by the men themselves (i.e., excluding questionnaires completed by next of kin). More White men correctly identified ageing as an important cause of prostate cancer (*P*<0.001), whereas just under half of both Black men and White men correctly identified family history as an important cause. About two-thirds of both Black men and White men correctly omitted smoking from their list of risk factors. Several dietary components are suspected risk factors for prostate cancer, and thus with some justification around a one-third of both Black men and White men identified bad diet as a risk factor. With similar justification, a small number of men identified obesity as a risk factor, with strong evidence that White men were more likely to select this factor (*P*=0.002). Black men were more likely to identify chemical exposure (*P*=0.026) and infection (*P*=0.041), although convincing evidence is yet to result from studies of these factors (Van Maele-Fabry and Willems, 2003; Wagenlehner *et al*, 2007). Few men volunteered other causes, notably race, which was missing from the list provided. Further into the questionnaire men were asked whether there was a difference in prostate cancer risk between Black men and White men and if so which men were at higher risk. Black men were less likely to take the ‘do not know’ option allowed for this question, and were more likely to know that Black men are at higher risk (*P*<0.001; Table 1).

In 1997, there were 54 new cases of prostate cancer for every 10 000 men aged 65 or over in the United Kingdom (www.cancer.org.uk), Black men and White men being equally prone to overestimate this figure (Table 1). More Black men correctly stated lung cancer to be more or equally common to prostate cancer (*P*=0.033), whereas more White men were correct in stating that testicular cancer was less common than prostate cancer (*P*<0.001; male cancer incidences per 100 000 person years in 1997 were 70 for prostate cancer, 77 for lung cancer; and 6 for testicular cancer).

Information on any delay between the appearance of symptoms and first presentation to a doctor was collected from hospital records and through an item in the questionnaire. There was 84% agreement for the 91 men with information from both sources, and as the questionnaire data and hospital records data supported identical conclusions, only the former are presented (Table 2 ). There was no convincing evidence of Black men delaying their presentation more than White men with more than half of all men seeking attention within 3 months of symptoms developing. More than 95% of all men were able to provide at least one reason for their delayed presentation, with Black men more likely to report each of the reasons in Table 2. Once differences in age were accounted for, Black men were more likely to be concerned that their symptoms may be due to something serious (*P*<0.001), were more embarrassed by their symptoms (*P*=0.009), and were more likely to dislike seeing their doctor in general (*P*=0.030).

Black men and White men were equally likely to present with co-morbid conditions (Table 3 ). Around 10% of men of both races presented with symptoms suggestive of metastases, and around two-thirds of all men presented with lower urinary tract symptoms (LUTS), predominantly storage and voiding symptoms (Table 3). There was weak evidence that Black men were more likely to be diagnosed in the absence of symptoms following a PSA test (*P*=0.052), although this association was attenuated after accounting for the age difference.

Referrals to a specialist for diagnosis, an urologist in 97% of cases, came from general practitioners, accident and emergency departments, and other hospital departments. Age- and centre-adjusted multinomial logistic regression analyses provided evidence that the presence of symptoms (*P*<0.001) and race (*P*=0.013) were independently associated with the source of the referral (Table 4 ). In particular, although general practitioners were by far the most common source of referral for all men, Black men were more likely than White men to be referred by the emergency department (odds ratio 1.66, 95% CI: 1.02–2.70) or another hospital team (odds ratio 1.67, 95% CI: 1.17–2.39). An age- and centre-adjusted logistic regression analysis, including referral source, race, and symptoms as covariates, indicated that referral source was associated with diagnosis as an inpatient (*P*<0.001) but that symptoms at diagnosis were not (*P*=0.25). Evidence of Black men being less likely to be diagnosed as inpatients was apparent in a centre-adjusted analysis (*P*=0.011), but the association was largely explained by the younger age of the Black men (age- and centre-adjusted odds ratio=0.75, 95% CI: 0.49–1.15; *P*=0.19).

A pretreatment PSA level at diagnosis was available for 458 Black men and 921 White men. The level was 10 ng ml^−1^ or less for 121 (26%) Black men and 226 (25%) White men, was more than 10 ng ml^−1^ but no more than 20 ng ml^−1^ for 79 (17%) Black men and 199 (22%) White men, and was more than 20 ng ml^−1^ but no more than 100 ng ml^−1^ in 163 (36%) Black men and 303 (33%) White men. A centre-adjusted comparison of these PSA levels between Black men and White men provided no convincing evidence of a difference (ordered logistic regression odds ratio 1.14, 95% CI: 0.92–1.41, *P*=0.23), but there was strong evidence of higher levels in Black men when differences in age at diagnosis were accounted for (odds ratio 1.59, 95% CI: 1.27–1.99, *P*<0.001).

## Discussion

In this cohort of men in southern England, Black men were diagnosed with prostate cancer at a younger age than White men, lived in less affluent areas and were more likely to have been in a manual occupation. Knowledge of prostate cancer was comparable, with White men more likely to know of age as a risk factor and Black men more likely to know of their own higher risk of developing the disease. There was no evidence of Black men or White men being more likely to delay presentation with symptoms, although Black men reported more reasons if they had delayed presentation. At diagnosis, Black men and White men were equally likely to have co-morbidity, symptoms of metastatic prostate cancer, and LUTS. There was weak evidence that Black men were more likely to have had their PSA level measured in the absence of symptoms, although this difference appeared to be due to the younger age of the Black men at diagnosis. Compared with White men of the same age, Black men were more likely to have been referred for diagnosis by a hospital-based team. At the time of diagnosis, Black men and White men had comparable PSA levels, although evidence of higher levels in Black men emerged when compared with their same-age White counterparts.

Our finding that Black men are diagnosed 5 years earlier than White men is in part explained by differences in the study population age distributions of these two groups. This is not a complete explanation as there is evidence of an interaction between race and age in the PROCESS study cohort (*P*<0.001), with the higher relative rates for Black men compared with White men being more marked for the younger age groups (Ben-Shlomo *et al*, 2008). This interaction was not found for the US-based Health Professionals Follow-up Study (Giovannucci *et al*, 2007), but analysis of US SEER data still finds Black men to be diagnosed when an average of 3 years younger than White men (Karami *et al*, 2007), in the absence of marked racial differences in age distribution in the United States.

It is possible that Black men are developing prostate cancer at the same age on average as White men but are being diagnosed earlier, this partially explaining the apparently higher incidence rate in Black men. This explanation is unlikely for the following reasons: (i) Black men were more likely to be in less affluent socioeconomic positions, such positions being associated with poorer access to health-care services (Nazroo, 1997); (ii) although Black men are more likely than White men to know of their own higher risk for prostate cancer, this knowledge was far from widespread in the study population, as has been observed elsewhere (Schulman *et al*, 2003); (iii) there was no evidence that Black men sought medical attention earlier for their symptoms, in line with data from the ‘National Survey of NHS patients: Cancer’ (Neal and Allgar, 2005); (iv) it is unlikely that Black men were subject to a higher rate of incidental detection, as they were no more likely to have co-morbidities or LUTS, and a higher chance of having been referred for diagnostic investigation by a hospital department is in the context of the large majority of all men having been referred by their GP. There was weak evidence of more Black men being diagnosed in the absence of symptoms following a PSA test, but this is largely explained by higher rates of PSA testing in younger men in the study population. In any case PSA levels, and the prevalence of symptoms suggestive of metastases, are comparable between Black men and White men, both these factors suggesting diagnosis at a similar stage of the disease. Consequently, the diagnosis of prostate cancer in Black men at an earlier age may be due to a greater biological susceptibility to the disease.

Our study is limited by its retrospective design, and the consequent reliance on extracting data from routine medical records and on the information recalled by men when completing the questionnaires. However, although the completeness and accuracy of measurements derived from routine records is of general concern, there is no reason to expect differences in record keeping practice for Black patients and White patients to introduce bias into our results. The detailed questionnaire may have been off-putting for some men, and it may be that those men who failed to return the questionnaire are more likely to have difficulty accessing health-care services (Wolf *et al*, 2006). Consequently measures derived from the questionnaire should be interpreted with this possible bias in mind.

In conclusion, differences in knowledge of prostate cancer, co-morbidity, testing rates, presentation following the appearance of symptoms, and referral pathways, are modest and seem insufficient to explain the higher rate of prostate cancer diagnosis in Black men compared with their White counterparts. We found no evidence that Black men had better or worse access to diagnostic services, our data being consistent with a genuinely higher prostate cancer incidence rate in Black men. Comparable PSA levels between Black men and White men suggest that diagnosis occurs at a similar disease stage; future studies of the PROCESS cohort will ascertain whether clinical management and prognosis are also comparable.

## Figures and Tables

**Table 1:** Knowledge of prostate cancer by race in PROCESS cohort members who completed the questionnaire themselves (i.e., excluding questionnaires completed by next of kin)

	**Black men *n* (%)**	**White men *n* (%)**	***P*-value^a^**
*Three most important causes of prostate cancer?*			
Men responding with at least one cause	179	453	
Ageing	119 (66)	368 (85)	<0.001
Family history	81 (45)	160 (37)	0.051
Smoking	58 (32)	159 (37)	0.33
Bad diet	56 (31)	153 (35)	0.36
Infection	47 (26)	82 (19)	0.041
Chemical exposure	41 (23)	67 (15)	0.026
Occupation	24 (13)	51 (12)	0.56
Obesity	6 (3)	48 (11)	0.002
Where you live	13 (7)	19 (4)	0.14
Other	9 (5)	27 (6)	0.57
			
*Comparing Black and White men*			
Black men have greater risk for prostate cancer	44 (23)	62 (13)	
White men have greater risk for prostate cancer	0 (0)	13 (3)	
No difference in risk	80 (42)	132 (29)	
Do not know	65 (34)	242 (54)	<0.001
			
*10 000 men over 65 years of age, how many new cases of prostate cancer in a 1-year period?*			
1	1 (1)	0 (0)	
10	10 (6)	12 (3)	
100	53 (29)	112 (25)	
1000	59 (33)	173 (39)	
1001+	57 (32)	145 (33)	0.11
			
*In men, lung cancer is*			
More common than prostate cancer	50 (28)	100 (23)	
Equally common to prostate cancer	66 (37)	135 (31)	
Less common than prostate cancer	61 (34)	200 (46)	0.033
			
*Testicular cancer is*			
More common than prostate cancer	20 (13)	25 (6)	
Equally common to prostate cancer	47 (30)	77 (19)	
Less common than prostate cancer	90 (57)	313 (75)	<0.001

Counts are the number of men providing the specified response.

a *P*-values are obtained using a Pearson's *χ*^2^ test.

**Table 2:** Delay between start of symptoms and first presentation, and patient-reported reasons by race in PROCESS cohort members

			**Centre-adjusted**		**Age and centre adjusted**	
	**Black men *n* (%)**	**White men *n* (%)**	**Odds ratio (95% CI)**	***P*-value**	**Odds ratio (95% CI)**	***P*-value**
*Delay seeking medical attention (n=522)*						
Less than 1 month	50 (34)	126 (34)				
1–3 months	35 (24)	98 (26)				
4–6 months	28 (19)	51 (14)				
7–12 months	24 (16)	52 (14)				
1 or 2 years	5 (3)	27 (7)				
More than 2 years	4 (3)	22 (6)				
Odds ratio per category change			0.93 (0.66, 1.32)	0.69	0.82 (0.57, 1.19)	0.30
						
*At least one reason given (n=590)*	164	426				
Did not think symptoms were serious	79 (48)	169 (40)	1.38 (0.95, 2.02)	0.095	1.20 (0.80, 1.79)	0.37
Do not like seeing my own doctor in general	32 (20)	60 (14)	1.57 (0.95, 2.60)	0.076	1.81 (1.06, 3.10)	0.030
Was scared that it might be something serious	59 (36)	83 (19)	2.25 (1.48, 3.42)	<0.001	2.55 (1.62, 4.00)	<0.001
Found the symptoms an embarrassing problem	54 (33)	107 (25)	1.61 (1.06, 2.44)	0.024	1.81 (1.16, 2.81)	0.009

Data from questionnaire; 92 men reported not being able to remember the length of the delay, although most of these could provide a reason for the delay. Counts are the number of men providing the specified response.

**Table 3:** Co-morbidity, symptoms at diagnosis, and route to diagnosis in the absence of symptoms by race in PROCESS cohort members; information from hospital records

			**Centre adjusted**		**Age and centre adjusted**	
	**Black men *n* (%)**	**White men *n* (%)**	**Odds ratio (95% CI)**	***P*-value**	**Odds ratio (95% CI)**	***P*-value**
*Charlson co-morbidity score (n=1296)*						
0	257 (61)	494 (57)				
1	96 (23)	177 (20)				
2+	69 (16)	203 (23)				
Odds ratio per category change			0.95 (0.75, 1.22)	0.71	1.21 (0.94, 1.57)	0.14
						
*Hospital records review available (n=1467)*	474	993				
Symptoms of metastasis?	52 (11)	111 (11)	1.13 (0.78, 1.64)	0.51	1.32 (0.89, 1.96)	0.17
LUTS?	301 (64)	671 (68)	0.95 (0.74, 1.20)	0.66	1.02 (0.79, 1.31)	0.87
Storage symptoms?	232 (49)	537 (54)	0.96 (0.76, 1.21)	0.71	1.01 (0.79, 1.29)	0.96
Voiding symptoms?	227 (48)	537 (54)	0.88 (0.70, 1.11)	0.29	0.91 (0.71, 1.15)	0.42
Acute urinary retention?	55 (12)	113 (11)	1.04 (0.72, 1.49)	0.85	1.40 (0.95, 2.07)	0.093
Haematuria?	34 (7)	104 (10)	0.67 (0.44, 1.01)	0.056	0.72 (0.47, 1.12)	0.15
Urinary tract infection?	35 (7)	62 (6)	1.19 (0.76, 1.85)	0.45	1.49 (0.92, 2.42)	0.10
Chronic urinary retention?	17 (4)	49 (5)	0.66 (0.37, 1.18)	0.17	0.83 (0.45, 1.53)	0.55
						
*Diagnosed in the absence of symptoms*						
With a PSA test	77 (16)	110 (11)	1.38 (1.00, 1.92)	0.052	1.26 (0.89, 1.77)	0.19
With digital rectal exam	18 (4)	34 (3)	1.07 (0.58, 1.96)	0.83	1.07 (0.57, 2.04)	0.83
With TURP	13 (3)	33 (3)	1.19 (0.58, 2.46)	0.64	1.59 (0.73, 3.46)	0.25

LUTS=lower urinary tract symptoms; PSA=prostate-specific antigen; TURP=trans-urethral resection of the prostate.

Counts are the number of men with the specified information in their hospital records.

**Table 4:** Pathways to diagnosis for Black and White men, with and without symptoms

	**Status at first appointment for diagnosis**		
**Source of referral**	**Outpatient (%)**	**In-patient (%)**	**Total**
*Symptomatic – Black men*			
General practitioner	214 (81)	7 (11)	221 (68)
Accident and emergency	9 (3)	25 (39)	34 (10)
Hospital team	25 (10)	32 (50)	57 (17)
Other	15 (6)	0 (0)	15 (5)
Total	263 (100)	64 (100)	327 (100)
			
*Symptomatic – White men*			
General practitioner	485 (85)	39 (27)	524 (74)
Accident and emergency	10 (2)	43 (30)	53 (7)
Hospital team	38 (7)	58 (40)	96 (13)
Other	35 (6)	4 (3)	39 (5)
Total	568 (100)	144 (100)	712 (100)
			
*No symptoms – Black men*			
General practitioner	65 (67)	2 (17)	67 (61)
Accident and emergency	2 (2)	3 (25)	5 (5)
Hospital team	18 (19)	6 (50)	24 (22)
Other	12 (12)	1 (8)	13 (12)
Total	97 (100)	12 (100)	109 (100)
			
*No symptoms – White men*			
General practitioner	77 (65)	5 (12)	82 (51)
Accident and emergency	1 (1)	5 (12)	6 (4)
Hospital team	23 (19)	15 (37)	38 (24)
Other	18 (15)	16 (39)	34 (21)
Total	119 (100)	41 (100)	160 (100)

Data from hospital records. Counts are the number of men with the specified combination of information in their hospital records.

## Appendix

The PROCESS study group included Bristol (P Abrams, D Dickerson, S Falk, R Feneley, D Gillatt, C Gingell, J Graham, F Keeley, H Newman, R Persad, J Probert, H Schwaibold, G Sibley, A Timoney, R Wells, T Whittlestone, M Wright), SW London (C Anderson, K Anson, M Bailey, R Kirby) SE London (D Cahill, P Dasgupta, J Glass, S Khan, G Muir, T O’Brien, R Popert, J Poulsen, P Thompson, R Tiptaft, K Walsh), NE London (N Buchholz, F Chinegwundoh, C Fowler, I Junaid, V Nargund, A Paris, J Pati,), Sheffield University (D Dorling B Thomas).
